# Seed biopriming with *Bacillus nematocida* enhances drought tolerance in maize via regulation of stress-responsive genes

**DOI:** 10.1038/s41598-025-29329-z

**Published:** 2025-12-05

**Authors:** Nermin G. Mohamed, Asmaa Mokhtar, Youmna Khaled, Nada Mohamed, Alaa Mahmoud

**Affiliations:** 1 Department of Agricultural Biotechnology, College of Biotechnology, Misr University of Science and Technology, Giza, 3236101 Egypt; 2 College of Biotechnology, Misr University of Science and Technology, Giza, 3236101 Egypt

**Keywords:** Maize, Drought stress, Seed biopriming, *Bacillus nematocida*, Stress-responsive genes, Expression systems, Plant biotechnology

## Abstract

Maize (*Zea mays* L.), a globally important cereal, is highly vulnerable to climate-induced drought stress. This study evaluated seed biopriming with *Bacillus nematocida* as a strategy to enhance drought tolerance through molecular reprogramming. Maize seeds were bioprimed and subjected to drought stress under greenhouse conditions, and the expression of twelve drought-responsive genes was analyzed via RT-qPCR. Biopriming strongly upregulated stress-related genes, including *PLD* (60-fold), *PYL1* (63-fold), *SLAH1* (11-fold), and *OST1* (7-fold) under combined drought + biopriming, while *Peroxidase* and *ZmPP2CA* were markedly suppressed. These transcriptional shifts indicate enhanced ABA signaling, ion homeostasis, and reduced oxidative stress load. Overall, *Bacillus nematocida* biopriming conferred a > 50-fold activation of key drought regulators, highlighting its potential as a sustainable approach to improve maize resilience under water-limited conditions.

**Supplementary Information:**

The online version contains supplementary material available at 10.1038/s41598-025-29329-z.

## Introduction

Maize (*Zea mays* L.), often referred to as the “queen of cereals,” is a vital crop with global significance, not only for food security but also for numerous industrial applications. It serves as a primary dietary and economic resource for a significant portion of the world’s population^1^. As one of the most widely cultivated cereal crops, maize ranks third globally in production, following rice and wheat, owing to its high nutritional value and rich phytochemical composition^2–4^.

However, climate change, particularly global warming, poses a serious threat to agricultural productivity and food security worldwide^5^. Maize production is highly sensitive to environmental conditions and is primarily grown in semi-arid and semi-humid regions^6^. Increasingly erratic climatic patterns have intensified the occurrence of abiotic stresses such as drought, heat, salinity, waterlogging, and cold which severely affect maize growth and yield. However, the drastic climate change scenario induces various abiotic stresses, including drought, heat, salinity, waterlogging, and cold stress^7^. Drought is one of the most severe stresses, reducing crop productivity and leading to large economic losses^8^. Drought stress triggers the overproduction of reactive oxygen species (ROS), including hydroxyl radicals, hydrogen peroxide, and superoxide anions, which disrupt cellular processes and damage plant tissues, ultimately impairing growth and development^9,10^. Maize’s susceptibility to drought highlights the urgent need to improve its resilience in water-restricted environments. Seed enhancement techniques such as priming, humidification, and presoaking have been reported to improve germination, seedling vigor, and yield under stress conditions. Additional strategies, including seed coating, chemical treatments, and microbial inoculation, also contribute to enhanced plant performance^11^. Seed priming, defined as a controlled pre-sowing hydration technique, has attracted increasing attention in recent years due to its proven ability to enhance seedling vigor, accelerate germination, and improve plant resilience under diverse stress conditions. Biopriming, which integrates priming with beneficial microbial inoculation, further enhances plant resilience by stimulating physiological and molecular stress responses^12,13^. Biostimulants used in biopriming may include humic and fulvic acids, chitosan, algal extracts, and various plant growth-promoting microorganisms^14^. Notably, the use of beneficial bacteria such as *Bacillus nematocida* in seed biopriming has shown promising potential in mitigating drought stress and improving crop performance^15^. Although several Bacillus species such as *Bacillus subtilis* and *Bacillus amyloliquefaciens* have been extensively studied for their role in enhancing drought tolerance in maize^16^, little attention has been given to *Bacillus nematocida*. This strain is particularly unique due to its strong root-colonization capacity, production of diverse secondary metabolites, and potential dual role in promoting plant growth while simultaneously mitigating biotic and abiotic stress. While *Bacillus subtilis* and *Bacillus amyloliquefaciens* have been widely used as seed-biopriming agents for maize under abiotic stress, their mechanisms are largely confined to phytohormone modulation, biofilm formation, and nutrient mobilization. In contrast, *Bacillus nematocida* exhibits a distinct trait set, including potent root colonization, secretion of volatile ketones such as 6-methyl-2-heptanone, and dual abiotic/biotic stress mitigation capacity. These characteristics support its mechanistic novelty and applied promise in maize drought-stress amelioration^17^. Here we present, for the first time in maize drought-stress context, the gene expression modulations induced by *Bacillus nematocida* seed biopriming, and we propose that Bacillus nematocida seed biopriming triggers a distinct gene-regulation pattern in maize under drought stress, indicating that this strain may activate unique signaling pathways and transcriptional networks compared with other *Bacillus* species. These distinctive features suggest that *Bacillus nematocida* may confer drought tolerance through mechanisms that differ from or complement those reported for other *Bacillus* strains, thereby offering a novel perspective in microbial seed biopriming strategies. In this context, the present study aims to investigate the effects of seed biopriming using *Bacillus nematocida* and the regulation of drought-responsive genes to enhance drought tolerance in maize. These insights may contribute to developing sustainable strategies for maintaining maize productivity and ensuring food security under changing climatic conditions.

## Results

### Gene expression of drought responsive genes

**Fig. 1:**
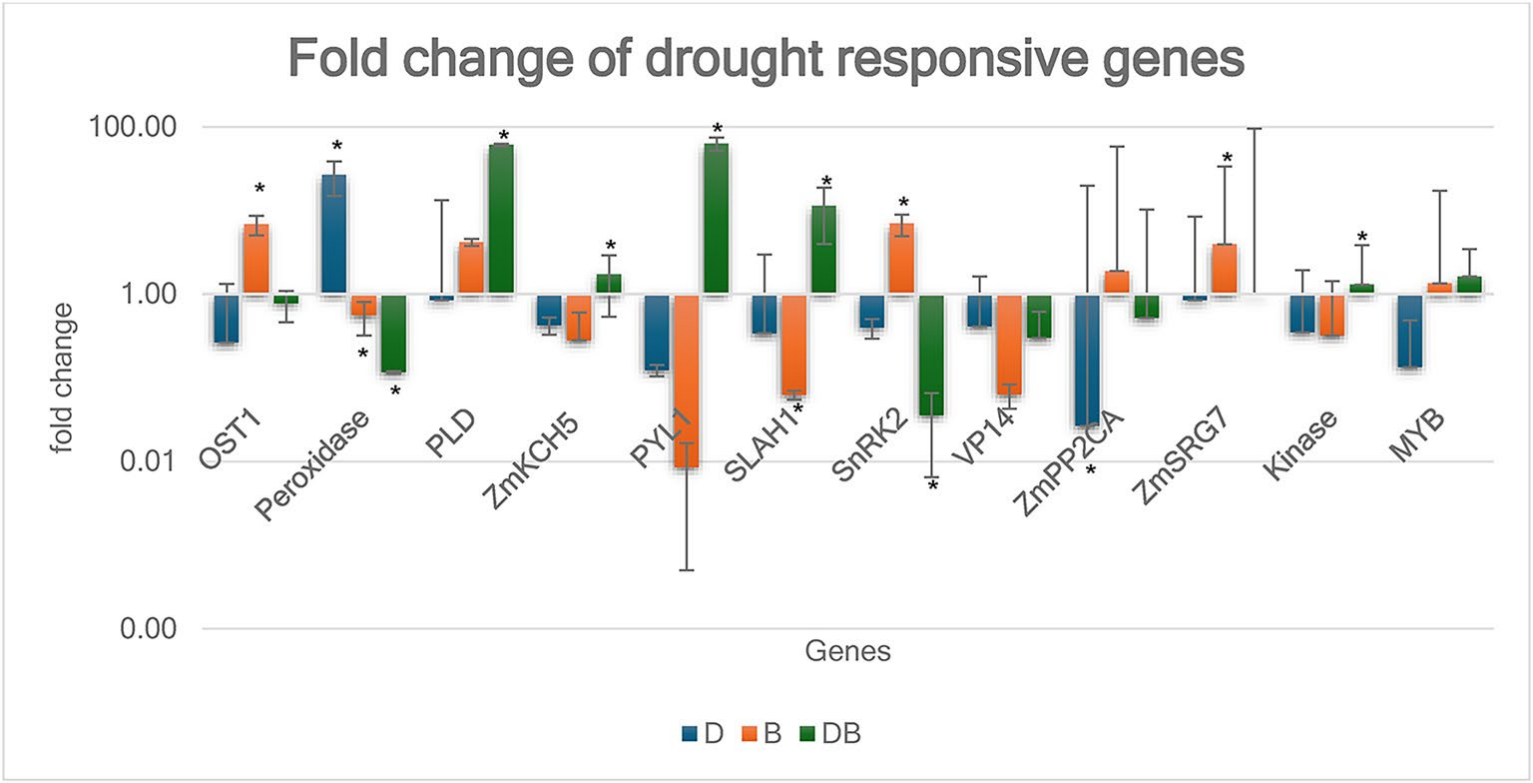
Fold change of drought-responsive genes in maize under different treatments. Bars represent the relative expression (fold change) of selected drought-responsive genes under drought (D), biopriming (B), and combined drought + biopriming (DB) treatments, relative to control. Data is shown as mean ± SD of three biological replicates. Error bars indicate standard deviation. * indicates significant differences based on Tukey’s HSD test at *P* ≤ 0.05.

To better understand the underlying regulatory mechanisms involved in maize drought tolerance, the expression of twelve stress-responsive genes was analyzed under four different treatments: Control (C), Drought (D), Biopriming with *Bacillus nematocida* (B), and Drought combined with Biopriming (DB). The expression data were grouped into five major functional categories reflecting key biological processes: ABA signaling, lipid signaling, antioxidant defense, transcriptional regulation, and ion transport and secondary signaling (Supplementary Table 1, Fig. 1).

### ABA signaling

Genes related to ABA signaling displayed contrasting patterns under the different treatments (Supplementary Table 1). The ABA receptor gene *PYL1* was strongly induced under drought combined with biopriming (62.97-fold), while remaining almost suppressed under drought (0.12-fold) and biopriming alone (0.01-fold). *OST1*, a central ABA signaling *kinase*, was highly upregulated under biopriming (6.84-fold), while showing only minor induction under drought (0.26-fold) and drought + biopriming (0.78-fold). In contrast, *SnRK2* exhibited strong induction under biopriming (6.96-fold) but was downregulated under drought (0.40-fold) and particularly under combined treatment (0.04-fold). The negative regulator *ZmPP2CA* showed modest induction under biopriming (1.88-fold), while expression remained low under drought (0.03-fold) and drought + biopriming (0.52-fold). Interestingly, the ABA biosynthesis gene *VP14* was repressed in all treatments, with fold changes below 0.5, suggesting that priming-enhanced ABA signaling relies more on perception and downstream regulation rather than biosynthesis.

### Lipid signaling

Among the tested genes, *PLD* demonstrated a highly dynamic expression profile, especially under DB treatment, Phospholipid signaling showed a remarkable treatment-specific pattern. *PLD* was strongly upregulated under combined drought + biopriming (60.83-fold) compared with modest induction under biopriming alone (4.17-fold), while remaining repressed under drought (0.84-fold). This indicates that lipid-mediated signaling plays a central role in the synergistic response to combined stress.

### Antioxidant pathways

The *Peroxidase* gene showed a strong drought-specific induction (26.72-fold), while it was markedly downregulated under biopriming (0.56-fold) and drought + biopriming (0.12-fold). These results suggest that canonical *peroxidase*-based ROS scavenging is mainly activated under drought, whereas biopriming redirects antioxidant responses away from peroxidase activity.

### Transcriptional regulators

Transcriptional regulators exhibited priming-associated activation. *MYB* expression was suppressed under drought (0.13-fold) but induced under biopriming (1.34-fold) and further increased under combined treatment (1.62-fold). Similarly, *ZmSRG7* was moderately upregulated by biopriming (3.94-fold), while showing almost no induction under drought (0.84-fold) and combined stress (1.00-fold). These patterns suggest that transcriptional reprogramming is more strongly triggered by biopriming than by drought alone.

### Ion transport and secondary signaling

Ion transport and secondary signaling genes showed treatment-specific modulation. *SLAH1*, encoding an S-type anion channel, was moderately induced under combined treatment (11.34-fold), but strongly suppressed under drought (0.34-fold) and biopriming (0.06-fold). Potassium channel 5 *(ZmKCH5)* showed low fold change under drought (0.43-fold) and biopriming (0.28-fold) but was modestly induced under combined treatment (1.72-fold). Similarly, *Kinase* showed slight repression under drought (0.35-fold) and biopriming (0.32-fold), with modest upregulation under combined stress (1.30-fold).

## Discussion

The present study demonstrates that biopriming with *Bacillus nematocida* substantially modulates the expression of drought-responsive genes in maize, leading to distinct transcriptional reprogramming compared to drought stress alone. Several key regulators exhibited unique expression patterns, highlighting the role of biopriming as a potential strategy to mitigate abiotic stress through priming-induced molecular memory. These findings are consistent with earlier reports showing that Bacillus-based seed priming enhances crop stress tolerance by reprogramming physiological and molecular responses^15,18^. A particularly striking feature of our dataset was the synergistic induction of *PYL1* and *OST1* under biopriming with *Bacillus nematocida* indicating potential amplification of ABA perception. ABA receptors (*PYL*/*RCAR* family) and the *OST1 kinase* form the core of stomatal closure signaling^19^. Their combined induction suggests that *B. nematocida* biopriming enhances ABA receptor sensitivity while simultaneously activating downstream phosphorylation cascades, leading to more efficient stomatal regulation. Previous studies have shown that microbial inoculants can fine-tune ABA signaling by enhancing receptor sensitivity and reducing dependence on de novo ABA synthesis^20–22^, which aligns with our findings.

Interestingly, *SnRK2*, a central ABA-activated *kinase*, was strongly induced under biopriming alone but suppressed in the combined treatment. This suggests that priming prepares the plant via a “molecular memory” mechanism that reduces the need for excessive *kinase* activation under actual drought stress. Similar observations of stress memory, where priming dampens transcriptional amplitude to balance energy use, have been reported^20–25^. It is important to note, however, that these interpretations remain hypothetical and based on transcriptional patterns. Further physiological and biochemical validation is required to confirm whether such molecular memory and energy-efficient responses translate into functional drought tolerance.

The expression of *VP14*, a key carotenoid cleavage dioxygenase in ABA biosynthesis, was moderately enhanced under biopriming but reduced under drought + biopriming. *VP14* encodes the 9-cis-epoxycarotenoid dioxygenase (*NCED*) responsible for a rate-limiting step in ABA biosynthesis^26,27^. Its downregulation under combined stress suggests that bioprimed plants may rely more on enhanced ABA perception (via *PYL1*/*OST1*) rather than increased biosynthesis, thus conserving resources while maintaining effective stress signaling.

In contrast to studies reporting sustained *peroxidase* induction under *Bacillus* priming^28^, our results showed significant repression of peroxidase transcripts in bioprimed plants. This indicates a shift away from classical ROS detoxification toward alternative antioxidant and signaling pathways. Supporting this, both *PLD* and *SLAH1* were significantly upregulated under drought + biopriming. *PLD*-mediated lipid signaling and *SLAH1*-dependent anion transport are key for regulating stomatal closure and water-use efficiency^29,30^.

The regulation of Potassium channel 5 (*ZmKCH5*) also revealed an important mechanism: the gene was suppressed under drought and biopriming individually but strongly induced under combined treatment. Potassium channels are essential for osmotic adjustment and maintaining turgor pressure under water deficit^31,32^. Thus, biopriming appears to restore potassium transport capacity under stress, improving osmotic balance and cell hydration.

The induction of *ZmPP2CA* and *ZmSRG7* further illustrates the complexity of priming responses. *ZmPP2CA*, a negative regulator of ABA signaling, showed moderate induction, likely serving as a feedback mechanism to prevent runaway ABA responses and maintain homeostasis^23,33^. *ZmSRG7*, which has been linked to stress adaptation, was significantly upregulated under biopriming, suggesting roles in osmolyte regulation and protective metabolite accumulation under biopriming.

The induction of *MYB* transcription factors under biopriming also highlights their importance in orchestrating drought responses. *MYB*s regulate osmolyte accumulation, cuticle formation, and activation of late stress-responsive genes^34^. Their induction suggests that *B. nematocida* activates broad transcriptional reprogramming in maize.

Finally, kinase-like transcripts showed moderate induction, which may reflect additional signaling cascades complementary to ABA. *Kinase*-mediated signaling plays critical roles in integrating ABA, ROS, and ion signaling during stress^19^. The *kinase* significantly induced under drought + biopriming which suggests that biopriming fine-tunes kinase activity, preventing excessive activation while maintaining signaling flexibility.

Collectively, these results demonstrate that *Bacillus nematocida* biopriming reshapes maize drought responses by (i) amplifying ABA perception and signaling (*PYL1*, *OST1*), (ii) modulating ABA biosynthesis (*VP14*), (iii) shifting antioxidant strategies away from peroxidase toward lipid- and ion-mediated signaling (*PLD*, *SLAH1*), (iv) restoring potassium transport (*ZmKCH5* ), and (v) broadly reprogramming transcriptional networks (*MYB*, *ZmSRG7*). It is important to note that the current findings represent transcriptional indications of enhanced drought tolerance rather than direct physiological confirmation. Future studies integrating physiological and biochemical measurements (e.g., photosynthetic efficiency, relative water content, or biomass) are warranted to validate the functional outcomes of the observed gene-regulation patterns. This multilayered regulatory adjustment underscores microbial priming as a powerful and sustainable strategy for improving crop drought resilience.

## Methods

### Bacterial culture

*Bacillus nematocida* was initially cultured on nutrient agar (NA) medium to ensure purity and isolate single colonies. The NA plates were incubated at 30 °C for 24 h. A single, well-isolated colony was then aseptically transferred into 50 mL of sterile Luria-Bertani (LB) broth in a 250 mL Erlenmeyer flask. The bacterial culture was incubated at 30 °C with constant agitation at 150 rpm for 16–18 h to achieve exponential phase growth.

The optical density (OD) of the culture was measured at 600 nm using a spectrophotometer, and the bacterial suspension was adjusted to an OD600 of 0.8 (approximately 10⁸ CFU/mL) to standardize the bacterial concentration for subsequent seed biopriming experiments. The prepared suspension was stored at room temperature and used immediately.

### Biopriming and growth conditions

Maize seeds of the drought-sensitive cultivar SC180 were obtained from the Maize Research Department, Field Crops Research Institute, Agricultural Research Center (ARC), Egypt. Uniform seeds were surface sterilized by immersion in 1% sodium hypochlorite solution for 5 min, followed by five rinses with sterile distilled water to remove any residual sterilizing agent. The sterilized seeds were then bioprimed by soaking in a suspension of *Bacillus nematocida* at room temperature for 2 h. After biopriming, the seeds were air-dried under aseptic conditions on sterile filter paper for 12 h.

### Drought stress treatment

The treated seeds were sown in autoclaved pots (25 cm diameter) filled with a loamy soil-perlite mixture (3:1, v/v). Pots were maintained in a greenhouse under controlled conditions: natural light with a 16/8 h light/dark photoperiod, temperature of 25 ± 2 °C, and relative humidity of 60–70%.

Drought stress treatment was initiated 45 days after sowing, during the vegetative stage, when plants had developed three fully expanded trifoliate leaves. Field capacity (FC) was determined gravimetrically by weighing pots after irrigation and allowing free drainage for 24 h, with the recorded weight representing 100% FC. Drought stress was imposed by withholding irrigation for 21 days, during which soil moisture declined to approximately 25–30% of FC. Control plants were maintained at 100% FC throughout the experiment. At the end of the stress period, plant samples were harvested and immediately flash-frozen in liquid nitrogen for subsequent RNA extraction.

### Primer design

Primers for quantitative gene expression analysis were designed using Primer-BLAST available from the National Center for Biotechnology Information (NCBI). Target-specific primers were selected based on the annotated gene sequences and optimized for specificity and amplification efficiency (Supplementary Table 2).

### RNA extraction and RT-PCR

Total RNA was extracted from the leaves using the COL-NA isolation kit (REME-D, Cairo, Egypt; Lot No. DE1-24-014) following the manufacturer’s protocol, and RemeMixgreen plus One-Step RT-qPCR Master Mix (REME-D, Cairo, Egypt; Lot No. DR06-25-002) was used for qPCR. The PCR procedure involved a reverse transcription at 45 ◦C for 20 min, hotstart activation at 94 ◦C for 2–5 min, followed by 40 cycles for amplification (94 °C for 10 s and 50–60 °C for 30 s).

### qPCR data analysis

The gene expression experiments were done in three biological replicates. RT-PCR data were analyzed by the relative quantification 2^−ΔΔCt^ method^35^. The relative difference in gene expression using the 2^−ΔΔCt^ method was calculated as follows: (*Δ*Ct = Ct target gene - Ct housekeeping gene, ΔΔCt = Ct treatment - Ct control, Relative fold change in gene expression = 2^−ΔΔ*Ct*^*)*, (Supplementary Table S1)

### Statistical analysis

The gene expression experiments were done in three replicates. All data were subjected to statistical analysis using the MSTATC software (Michigan State University, East Lansing, MI, USA). Analysis of variance (ANOVA) was performed to evaluate the significance of differences among treatments, and means were compared using the Tukey’s Honestly Significant Difference test at a 5% probability level. A P-value ≤ 0.05 was considered statistically significant.

## Conclusion

The findings of this study confirm that seed biopriming with *Bacillus nematocida* can effectively enhance drought tolerance in maize by modulating stress-responsive gene expression. Under combined drought and biopriming treatment, significant upregulation of key ABA signaling genes and ion transporters, alongside altered antioxidant gene expression, highlights the multifaceted role of microbial priming in stress adaptation. This approach primes maize plants to respond more efficiently to water-deficit conditions, potentially improving crop performance and resilience in the face of increasing climatic challenges. Biopriming thus represents a practical and eco-friendly strategy to support sustainable maize cultivation under drought-prone environments.

## Supplementary Information

Below is the link to the electronic supplementary material.

Supplementary Material 1

Supplementary Material 2

## Data Availability

The gene-related datasets used in this study, including accession numbers, are provided in Supplementary Tables 2 and were obtained from the NCBI database. The nucleotide sequence used during the current study is available in the NCBI GenBank database under the following accession numbers: J01238: [https://www.ncbi.nlm.nih.gov/nuccore/J01238](https:/www.ncbi.nlm.nih.gov/nuccore/J01238)NM_001155730: [https://www.ncbi.nlm.nih.gov/nuccore/NM_001155730](https:/www.ncbi.nlm.nih.gov/nuccore/NM_001155730)NM_001112010.1: [https://www.ncbi.nlm.nih.gov/nuccore/NM_001112010.1](https:/www.ncbi.nlm.nih.gov/nuccore/NM_001112010.1)ONM53587.1: [https://www.ncbi.nlm.nih.gov/protein/ONM53587.1](https:/www.ncbi.nlm.nih.gov/protein/ONM53587.1)NM_001112432: [https://www.ncbi.nlm.nih.gov/nuccore/NM_001112432](https:/www.ncbi.nlm.nih.gov/nuccore/NM_001112432)NM_001367152: [https://www.ncbi.nlm.nih.gov/nuccore/NM_001367152](https:/www.ncbi.nlm.nih.gov/nuccore/NM_001367152)XM_008654699: [https://www.ncbi.nlm.nih.gov/nuccore/XM_008654699](https:/www.ncbi.nlm.nih.gov/nuccore/XM_008654699)NM_001130460: [https://www.ncbi.nlm.nih.gov/nuccore/NM_001130460](https:/www.ncbi.nlm.nih.gov/nuccore/NM_001130460)NM_001174602: [https://www.ncbi.nlm.nih.gov/nuccore/NM_001174602](https:/www.ncbi.nlm.nih.gov/nuccore/NM_001174602)NM_001399038: [https://www.ncbi.nlm.nih.gov/nuccore/NM_001399038](https:/www.ncbi.nlm.nih.gov/nuccore/NM_001399038)NM_001112216: [https://www.ncbi.nlm.nih.gov/nuccore/NM_001112216](https:/www.ncbi.nlm.nih.gov/nuccore/NM_001112216)NM_001157214: [https://www.ncbi.nlm.nih.gov/nuccore/NM_001157214](https:/www.ncbi.nlm.nih.gov/nuccore/NM_001157214)AJ401276: [https://www.ncbi.nlm.nih.gov/nuccore/AJ401276](https:/www.ncbi.nlm.nih.gov/nuccore/AJ401276).

## References

[1] Sheoran, S., Kumar, S., Kumar, P., Meena, R. S. & Rakshit, S. Nitrogen fixation in maize: breeding opportunities. *Theor. Appl. Genet.* **134**, 1263–1280 (2021). 33677701 10.1007/s00122-021-03791-5

[2] Yin, Z. et al. Differential responses of 23 maize cultivar seedlings to an arbuscular mycorrhizal fungus when grown in a metal-polluted soil. *Sci. Total Environ.* **789**, 148015 (2021). 34051499 10.1016/j.scitotenv.2021.148015

[3] Millet, E. J. et al. Genomic prediction of maize yield across European environmental conditions. *Nat. Genet.* **51**, 952–956 (2019). 31110353 10.1038/s41588-019-0414-y

[4] De Feudis, M., D’Amato, R., Businelli, D. & Guiducci, M. Fate of selenium in soil: A case study in a maize (Zea Mays L.) field under two irrigation regimes and fertilized with sodium selenite. *Sci. Total Environ.* **659**, 131–139 (2019). 30597463 10.1016/j.scitotenv.2018.12.200

[5] Guihur, A., Rebeaud, M. E. & Goloubinoff, P. How do plants feel the heat and survive? *Trends Biochem. Sci.* **47**, 824–838 (2022). 35660289 10.1016/j.tibs.2022.05.004

[6] Wang, F. et al. Grain yields and evapotranspiration dynamics of drip-irrigated maize under high plant density across arid to semi-humid climates. *Agric. Water Manage.* **247**, 106726 (2021).

[7] Gadag, R. et al. Resistance to biotic stress: theory and applications in maize breeding. *Genomic Designing Biotic Stress Resistant Cereal Crops*, 129–175 (2021).

[8] Wan, W. et al. Spatiotemporal patterns of maize drought stress and their effects on biomass in the Northeast and North China plain from 2000 to 2019. *Agric. For. Meteorol.* **315**, 108821. 10.1016/j.agrformet.2022.108821 (2022).

[9] Akyol, T. Y., YILMAZ, O., TÜRKAN, İ. & UZİLDAY, B., UZİLDAY, R. Ö. & Plant response to salinity: an analysis of ROS formation, signaling, and antioxidant defense. *Turkish J. Bot.* **44**, 1–13 (2020).

[10] Helal, N. M. et al. Improving yield components and desirable eating quality of two wheat genotypes using Si and NanoSi particles under heat stress. *Plants* **11**, 1819 (2022). 35890453 10.3390/plants11141819 PMC9316522

[11] Mitiku, T. Effect of Halo, osmo and Hydro-Priming on yield and yield related traits of common bean at Raya Valley of Tigray Region, Ethiopia. *J. Plant. Sci.* **11**, 74–79 (2023).

[12] Marthandan, V. et al. Seed priming: A feasible strategy to enhance drought tolerance in crop plants. *Int. J. Mol. Sci.* **21**10.3390/ijms21218258 (2020). 10.3390/ijms21218258 PMC7662356 33158156

[13] Saha, D. et al. Drought stress responses and inducing tolerance by seed priming approach in plants. *Plant. Stress*. **4**, 100066. 10.1016/j.stress.2022.100066 (2022).

[14] Malik, A. et al. Biostimulant-treated seedlings under sustainable agriculture: A global perspective facing climate change. *Agronomy* **11**, 14 (2020).

[15] Nawaz, H. et al. Seed biopriming mitigates terminal drought stress at reproductive stage of maize Byenhancing gas exchange attributes and nutrient uptake. *Turkish J. Agric. Forestry*. **44**, 250–261 (2020).

[16] Vardharajula, S., Zulfikar Ali, S., Grover, M., Reddy, G. & Bandi, V. Drought-tolerant plant growth promoting Bacillus spp.: effect on growth, osmolytes, and antioxidant status of maize under drought stress. *J. Plant Interact.* **6**, 1–14 (2011).

[17] Huang, X. W., Niu, Q. H., Zhou, W. & Zhang, K. Q. Bacillus nematocida sp. nov., a novel bacterial strain with nematotoxic activity isolated from soil in Yunnan, China. *Syst. Appl. Microbiol.* **28**, 323–327. 10.1016/j.syapm.2005.01.008 (2005). 15997705 10.1016/j.syapm.2005.01.008

[18] Aydinoglu, F., Kahriman, T. Y. & Balci, H. Seed bio-priming enhanced salt stress tolerance of maize (Zea Mays L.) seedlings by regulating the antioxidant system and MiRNA expression. *3 Biotech.* **13**, 378 (2023). 37900268 10.1007/s13205-023-03802-w PMC10600073

[19] Cutler, S. R., Rodriguez, P. L., Finkelstein, R. R. & Abrams, S. R. Abscisic acid: emergence of a core signaling network. *Annu. Rev. Plant Biol.* **61**, 651–679 (2010). 20192755 10.1146/annurev-arplant-042809-112122

[20] Lephatsi, M. et al. Molecular mechanisms associated with microbial biostimulant-mediated growth enhancement, priming and drought stress tolerance in maize plants. *Sci. Rep.* **12**, 10450 (2022). 35729338 10.1038/s41598-022-14570-7 PMC9213556

[21] Shaffique, S. et al. Seed Bio-priming of wheat with a novel bacterial strain to modulate drought stress in Daegu, South Korea. *Front. Plant Sci.* **14**, 1118941 (2023). 37180396 10.3389/fpls.2023.1118941 PMC10173886

[22] Shaffique, S. et al. Biopriming of maize seeds with a novel bacterial strain SH-6 to enhance drought tolerance in South Korea. *Plants* **11**, 1674 (2022). 35807630 10.3390/plants11131674 PMC9268940

[23] Fujii, H., Verslues, P. E. & Zhu, J. K. Identification of two protein kinases required for abscisic acid regulation of seed germination, root growth, and gene expression in Arabidopsis. *Plant. Cell.* **19**, 485–494 (2007). 17307925 10.1105/tpc.106.048538 PMC1867333

[24] Sah, S. K., Reddy, K. R. & Li, J. Abscisic acid and abiotic stress tolerance in crop plants. *Front. Plant Sci.* **7**, 571 (2016). 27200044 10.3389/fpls.2016.00571 PMC4855980

[25] Umezawa, T., Fujita, M., Fujita, Y., Yamaguchi-Shinozaki, K. & Shinozaki, K. Engineering drought tolerance in plants: discovering and tailoring genes to unlock the future. *Curr. Opin. Biotechnol.* **17**, 113–122 (2006). 16495045 10.1016/j.copbio.2006.02.002

[26] Tan, B. C., Cline, K. & McCarty, D. R. Localization and targeting of the VP14 epoxy-carotenoid dioxygenase to Chloroplast membranes. *Plant J.* **27**, 373–382 (2001). 11576422 10.1046/j.1365-313x.2001.01102.x

[27] Wan, X. R. & Li, L. Regulation of ABA level and water-stress tolerance of Arabidopsis by ectopic expression of a peanut 9-cis-epoxycarotenoid dioxygenase gene. *Biochem. Biophys. Res. Commun.* **347**, 1030–1038 (2006). 16870153 10.1016/j.bbrc.2006.07.026

[28] Lastochkina, O. et al. Seed priming with endophytic Bacillus subtilis modulates physiological responses of two different triticum aestivum L. cultivars under drought stress. *Plants* **9**, 1810 (2020). 33371269 10.3390/plants9121810 PMC7766295

[29] Gupta, A. & Rico-Medina, A. Caño-Delgado, A. I. The physiology of plant responses to drought. *Science* **368**, 266–269 (2020). 32299946 10.1126/science.aaz7614

[30] Zhu, J. K. Abiotic stress signaling and responses in plants. *Cell* **167**, 313–324 (2016). 27716505 10.1016/j.cell.2016.08.029 PMC5104190

[31] Anschütz, U., Becker, D. & Shabala, S. Going beyond nutrition: regulation of potassium homoeostasis as a common denominator of plant adaptive responses to environment. *J. Plant Physiol.* **171**, 670–687 (2014). 24635902 10.1016/j.jplph.2014.01.009

[32] Shabala, S. & Pottosin, I. Regulation of potassium transport in plants under hostile conditions: implications for abiotic and biotic stress tolerance. *Physiol. Plant.* **151**, 257–279 (2014). 24506225 10.1111/ppl.12165

[33] Xue, T. et al. Genome-wide and expression analysis of protein phosphatase 2 C in rice and Arabidopsis. *BMC Genom.* **9**, 550 (2008). 10.1186/1471-2164-9-550 PMC2612031 19021904

[34] Ambawat, S., Sharma, P., Yadav, N. R. & Yadav, R. C. MYB transcription factor genes as regulators for plant responses: an overview. *Physiol. Mol. Biology Plants*. **19**, 307–321 (2013). 10.1007/s12298-013-0179-1 PMC3715649 24431500

[35] Livak, K. J. & Schmittgen, T. D. Analysis of relative gene expression data using real-time quantitative PCR and the 2 – ∆∆CT method. *methods* 25, 402–408 (2001). 10.1006/meth.2001.1262 11846609

