# Supplementary material for: Seed biopriming with Bacillus nematocida enhances drought tolerance in maize via regulation of stress-responsive genes
Source: Sci Rep. 2025 Dec 5;15:43227. doi: 10.1038/s41598-025-29329-z (PMC12680607; doi:10.1038/s41598-025-29329-z)
Supplement: Supplementary file 1 — Supplementary Material 1 [file 41598_2025_29329_MOESM1_ESM.docx]

**Supplementary Table 1**. The fold change of drought-responsive genes in maize under different treatments.

|  | Drought | Biopriming | Drought + Biopriming |
| --- | --- | --- | --- |
| *OST1* | 0.26 | 6.84 | 0.78 |
| *Peroxidase* | 26.72 | 0.56 | 0.12 |
| *PLD* | 0.84 | 4.17 | 60.83 |
| *kch5* | 0.43 | 0.28 | 1.72 |
| *PYL1* | 0.12 | 0.01 | 62.97 |
| *SLAH1* | 0.34 | 0.06 | 11.34 |
| *SnRK2* | 0.40 | 6.96 | 0.04 |
| *VP14* | 0.41 | 0.06 | 0.30 |
| *ZmPP2CA* | 0.03 | 1.88 | 0.52 |
| *ZmSRG7* | 0.84 | 3.94 | 1.00 |
| *Kinase* | 0.35 | 0.32 | 1.30 |
| *MYB* | 0.13 | 1.34 | 1.62 |
