# Supplementary material for: Seed biopriming with Bacillus nematocida enhances drought tolerance in maize via regulation of stress-responsive genes
Source: Sci Rep. 2025 Dec 5;15:43227. doi: 10.1038/s41598-025-29329-z (PMC12680607; doi:10.1038/s41598-025-29329-z)
Supplement: Supplementary file 2 — Supplementary Material 2 [file 41598_2025_29329_MOESM2_ESM.docx]

**Supplementary Table 2.** The sequences of the designed primers for target and housekeeping genes.

| **Gene** | **Sequence 5'-3'** | **Accession no. of gene** |
| --- | --- | --- |
| *β-Actin* | F: GCCGATCGTATGAGCAAGGA  R: GGTCAAGGCTGCTGTAGGTT | J01238 |
| *MYB* | F: CCTAGAGAGCGCATGCAAGAT  R: GCCAACCTCAGAATTCGCTG | NM_001155730 |
| *kch5* | F: CCCACGATGCCAAACATCAA  R: CAAACTTCCTCGCGCCTAGT | NM_001112010.1 |
| *ZmSRG7* | F: CGGGAATGGSSGAAACCAAG  R: TTCATTGCCTACAAGATAGGC | ONM53587.1 |
| *VP14* | F: ACTGGGGTGTCTGGGATCTT  R: ATGTACACGCACCGATAGCC | NM_001112432 |
| *SLAH1* | F: AGACAGATAGAGCGACGACGAG  R: GCCATCGACGTGGGGTTTA | NM_001367152 |
| *Kinase* | F: TGCATTGGTTGGTAACAGAT  R: ATTGTGAATGGTACTGCCTT | XM_008654699 |
| *OST1* | F: AGAAGGCTCTTGTCTTCTACGC  R: CCTTGGGCACCGTCATCATCA | NM_001130460 |
| *SnRK2* | F: TGATGAGAACGTGCAGAGGG  R: AAAGCAGACTGAAAAGCACACT | NM_001174602 |
| *ZmPP2C-A* | F: TCCGCAAGGAAACTGACTCC  R: TGGATGAAAGGGGAAAGAAGAA | NM_001399038 |
| *PLD* | F: AAGGGAAAACTCTGCGAGGG  R: TCGCATCACCACTCTCACAC | NM_001112216 |
| *PYL1* | F: CGGCAAGGGAAGAAAGCAAC  R: TCACATGCCTCAAGATAGCCA | NM_001157214 |
| *Peroxidase* | F: CAACCTGGGCCTCTTCAAGT  R: AGTGAATCCTGATGGGCGG | AJ401276 |
